# BTB domain mutations perturbing KCTD15 oligomerisation cause a distinctive frontonasal dysplasia syndrome

**DOI:** 10.1136/jmg-2023-109531

**Published:** 2024-01-31

**Authors:** Kerry A Miller, David A Cruz Walma, Daniel M Pinkas, Rebecca S Tooze, Joshua C Bufton, William Richardson, Charlotte E Manning, Alice E Hunt, Julien Cros, Verity Hartill, Michael J Parker, Simon J McGowan, Stephen R F Twigg, Rod Chalk, David Staunton, David Johnson, Andrew O M Wilkie, Alex N Bullock

**Affiliations:** 1 MRC Weatherall Institute of Molecular Medicine, University of Oxford, Oxford, UK; 2 Centre for Medicines Discovery, University of Oxford, Oxford, UK; 3 Cell Biology Section, National Institute of Dental and Craniofacial Research, National Institutes of Health, Bethesda, Maryland, USA; 4 Department of Biological Sciences, Universidad Loyola Andalucía, Seville, Spain; 5 Leeds Institute of Medical Research, University of Leeds, Leeds, UK; 6 Yorkshire Regional Genetics Service, Leeds Teaching Hospitals NHS Trust, Chapel Allerton Hospital, Leeds, UK; 7 Sheffield Clinical Genomics Service, Sheffield Children's Hospital NHS Foundation Trust, Sheffield, UK; 8 Department of Biochemistry, University of Oxford, Oxford, UK; 9 Craniofacial Unit, Oxford University Hospitals NHS Foundation Trust, Oxford, UK

**Keywords:** Congenital, Hereditary, and Neonatal Diseases and Abnormalities, Exome Sequencing, Mutation, Missense, Structural Homology, Protein

## Abstract

**Introduction:**

*KCTD15* encodes an oligomeric BTB domain protein reported to inhibit neural crest formation through repression of Wnt/beta-catenin signalling, as well as transactivation by TFAP2. Heterozygous missense variants in the closely related paralogue KCTD1 cause scalp-ear-nipple syndrome.

**Methods:**

Exome sequencing was performed on a two-generation family affected by a distinctive phenotype comprising a lipomatous frontonasal malformation, anosmia, cutis aplasia of the scalp and/or sparse hair, and congenital heart disease. Identification of a de novo missense substitution within *KCTD15* led to targeted sequencing of DNA from a similarly affected sporadic patient, revealing a different missense mutation. Structural and biophysical analyses were performed to assess the effects of both amino acid substitutions on the KCTD15 protein.

**Results:**

A heterozygous c.310G>C variant encoding p.(Asp104His) within the BTB domain of *KCTD15* was identified in an affected father and daughter and segregated with the phenotype. In the sporadically affected patient, a de novo heterozygous c.263G>A variant encoding p.(Gly88Asp) was present in KCTD15. Both substitutions were found to perturb the pentameric assembly of the BTB domain. A crystal structure of the BTB domain variant p.(Gly88Asp) revealed a closed hexameric assembly, whereas biophysical analyses showed that the p.(Asp104His) substitution resulted in a monomeric BTB domain likely to be partially unfolded at physiological temperatures.

**Conclusion:**

BTB domain substitutions in KCTD1 and KCTD15 cause clinically overlapping phenotypes involving craniofacial abnormalities and cutis aplasia. The structural analyses demonstrate that missense substitutions act through a dominant negative mechanism by disrupting the higher order structure of the KCTD15 protein complex.

**WHAT IS ALREADY KNOWN ON THIS TOPIC**

- High throughput sequencing for craniofacial abnormalities has led to the identification of many causative genes.
- Two families presented with a clinically similar rare phenotype involving frontonasal mass and cutis aplasia or sparse hair of unknown cause.

**WHAT THIS STUDY ADDS**

- Phenotypes were linked to heterozygous amino acid substitutions in the BTB domain of *KCTD15*, resulting in perturbation of the normal pentameric assembly of the BTB domain.
- The *KCTD15* substitutions and phenotype clinically overlap with BTB domain substitutions in the paralogue *KCTD1*, which cause scalp-ear-nipple syndrome, consistent with partially overlapping functions.

**HOW THIS STUDY MIGHT AFFECT RESEARCH, PRACTICE OR POLICY**

- *KCTD15* is a new human disease gene that should be particularly scrutinised in patients presenting with frontonasal mass. The clinical features are consistent with developmental roles in the biogenesis of neural crest and skin.

## Introduction

The *KCTD15* gene has no current link to Mendelian disease, although flanking non-coding SNPs have been associated with obesity.^1 2^ Knockdown of the zebrafish orthologues *kctd15a* and *kctd15b* has demonstrated a role for KCTD15 in the development of the neural crest^3 4^ and for nephron segmentation in the kidney,^5^ and overexpression was associated with an attenuation of Wnt/beta-catenin signalling and repression of transactivation by the transcription factor TFAP2 (AP-2). Overexpression of KCTD15 has been reported in several cancers, including breast cancer and leukaemia.^6^ Silencing of *KCTD15* in cancer cell lines was antiproliferative and attenuated NF-κB signalling in models of *KMT2A*-rearranged leukaemia.^7 8^

KCTD15 is a member of the potassium (K^+^) channel tetramerisation domain (KCTD) family.^6 7 9^ All 25 members share an N-terminal BTB/POZ domain (abbreviated from broad-complex, tramtrack, and bric à brac, and poxvirus and zinc finger, respectively), with homology to the T1 tetramerisation domain of the voltage-gated potassium (Kv) channels, as well as a variable C-terminal domain (CTD). Despite the name, the KCTD family members are soluble non-channel proteins that typically assemble as homopentamers in the functional state.^10–15^ Many, if not all, have been shown to bind to Gβγ subunits to regulate G-protein-coupled receptor signalling,^14 16 17^ including some that assemble further into CUL3-dependent E3 ubiquitin ligases for Gβγ ubiquitination and degradation.^18^ However, KCTD15 lacks this common family interaction and its molecular mechanisms remain relatively poorly characterised.

**Figure 1:**
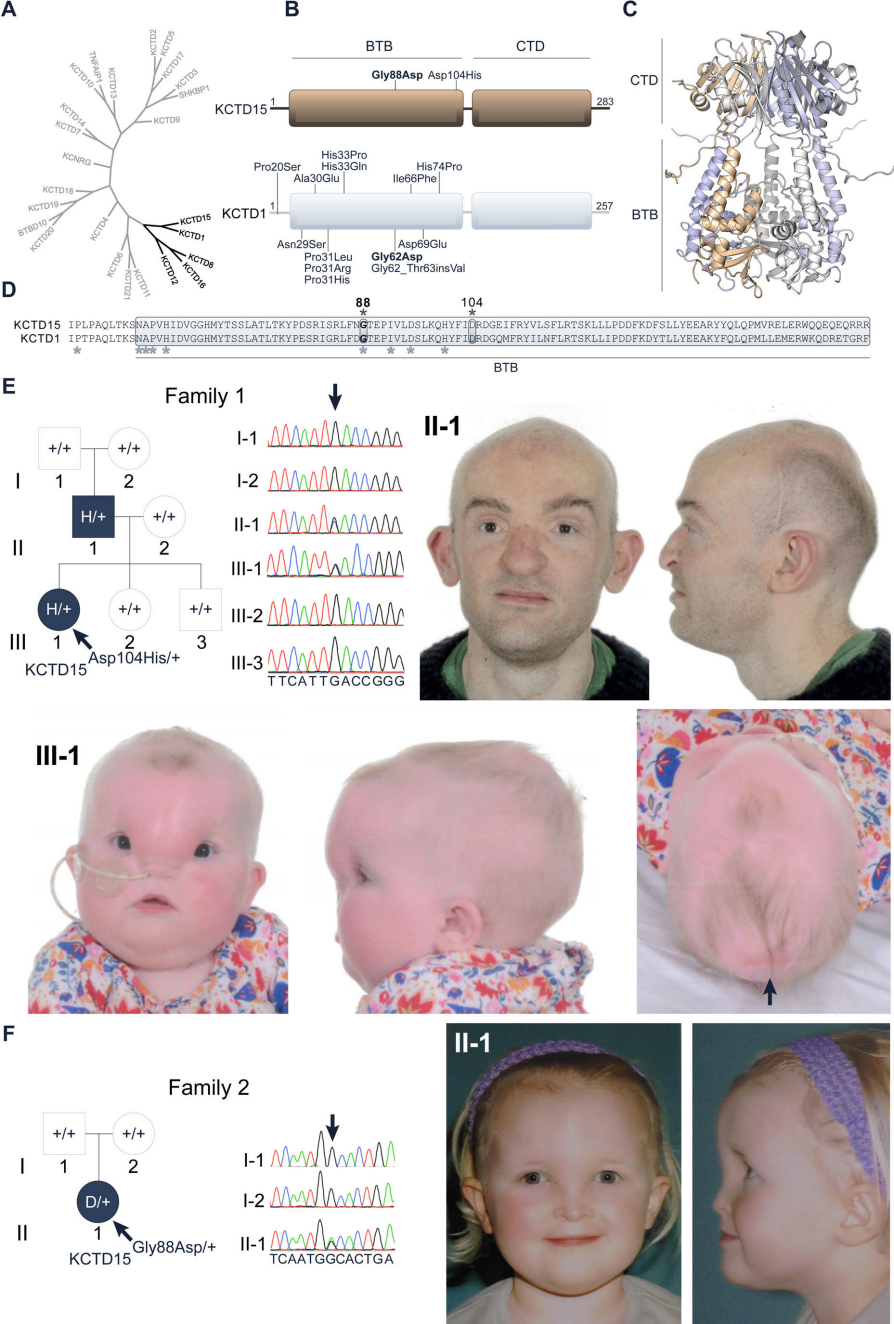
KCTD15 missense substitutions and associated clinical features. (A) Phylogenetic tree of the potassium (K^+^) channel tetramerisation domain (KCTD) family BTB domains. The cluster containing paralogues KCTD1, KCTD8, KCTD12, KCTD15 and KCTD16 is highlighted in black. (B) Domain organisations of KCTD15 and KCTD1 depicting the two novel de novo amino acid substitutions in KCTD15 causing a frontonasal dysplasia syndrome similar to scalp-ear-nipple (SEN) syndrome, and the 13 variants in KCTD1 causing SEN. The substitutions in KCTD15 and KCTD1 occurring at the equivalent positions (Gly88Asp and Gly62Asp, respectively) are shown in bold. (C) Homology model of a KCTD15 closed pentamer based on the KCTD1 structure (PDB ID 6S4L) showing the BTB and C-terminal domains (CTD). Different colours denote different KCTD15 subunits in the pentamer. (D) Sequence alignment of KCTD1 and KCTD15 BTB domains. The BTB domain is boxed and labelled and known substitution sites in KCTD15 and KCTD1 are marked with asterisks. (E) Analysis of Family 1, showing pedigree (left), dideoxy-sequencing of *KCTD15* exon 5 (centre) and craniofacial features of two affected family members (right and below). Photographs show the affected child III-1 before first birthday (preoperative) and her affected father II-1 in mid-30s (postoperative). Note hypertelorism and sparse hair present in both individuals; the child exhibits a large frontonasal lipoma and a region of aplasia cutis posteriorly located on the scalp (arrowhead). The sequence shows a heterozygous c.310G>C in II-1 and III-1 (arrow), which arose de novo in II-1. (F) Family 2 showing pedigree (left), dideoxy-sequencing of *KCTD15* exon 5 (centre) and preoperative craniofacial features of II-1 aged 2–4 years (right). Note hypertelorism and midline frontonasal swelling, which shows less lateral extension and nasal involvement compared with the proband in Family 1. The sequence chromatogram shows a heterozygous c.263G>A transition in II-1, absent in both her parents.

Structure predictions using AlphaFold cluster KCTD15 with the paralogues KCTD1, KCTD8, KCTD12 and KCTD16,^10^ consistent with primary sequence analyses (figure 1A). Within this group, KCTD15 is most closely related to KCTD1, with which it shares 80% sequence identity across its folded domains. Twelve distinct heterozygous missense substitutions and one in-frame insertion in the BTB domain of KCTD1 have been reported to cause the craniofacial condition scalp-ear-nipple (SEN) syndrome (figure 1B–D).^19–21^ Here, we report two de novo missense substitutions in the KCTD15 BTB domain that associate with a distinct frontonasal dysplasia as well as cutis aplasia or sparse hair reminiscent of the phenotypes observed in SEN. Structural and biophysical analyses show that these substitutions perturb BTB domain oligomerisation similarly to those identified in SEN to yield a deleterious effect.^20^

## Materials and methods

### Human genetic analysis

Genomic DNA was extracted from whole blood samples. For Family 1 we used a previously described protocol^22^ to capture human genomic DNA using the Agilent SureSelect v5 kit, followed by 100 bp paired-end sequencing using an Illumina HiSeq instrument. Reads were aligned to the GRCh38 reference genome using Bowtie2, and variant calling was performed using SAMtools v1.1 and Platypus v.0.5.2. We filtered for variants present in II-1, the proband’s father, but absent in either of his parents. The KCTD15 variant was confirmed by PCR amplification of exon 5 followed by dideoxy-sequencing (MRC Weatherall Institute of Molecular Medicine Sequencing Facility) and restriction digest using HphI. For Family 2 and additional patient samples analysed, the entire open reading frame of *KCTD15* was amplified and sequenced using the primers listed in online supplemental table S1. Reagents and conditions were used as previously described.^22^ Sample relationships in Family 2 were confirmed by a single molecule molecular inversion probe assay^23^ using a panel of 47 informative biallelic SNPs.

10.1136/jmg-2023-109531.supp1 Supplementary data

### KCTD15 protein expression and purification

Human KCTD15 BTB and BTB+CTD multidomain constructs (Uniprot Q96SI1, residues 52–165 and 52–283, respectively) were subcloned into the bacterial expression vector pGTVL2 (GenBank: JF522100.1) and transformed into BL21(DE3)-R3-pRARE cells. Cultures were grown in autoinduction Terrific Broth medium supplemented with 100 µg/mL kanamycin to an OD600 of 0.6 at 37°C and then cooled to 18°C for protein expression overnight. Cells were harvested by centrifugation, resuspended in binding buffer (50 mM HEPES pH 7.5, 500 mM NaCl, 5% glycerol, 5 mM imidazole) supplemented with Protease Inhibitor Cocktail Set III (Merck), 0.5 mM Tris(2-carboxyethyl)phosphine hydrochloride (TCEP) and lysed by sonication. DNA was precipitated from lysate using 0.15% polyethyleneimine and lysates were clarified by centrifugation for 60 min. GST-tagged proteins were immobilised on glutathione Sepharose overnight at 4°C then washed with binding buffer five times. Bound proteins were eluted by tag cleavage overnight at 4°C using TEV protease and then further purified by size exclusion chromatography (SEC) through a HiLoad 16/600 Superdex 200 preparatory grade column (GE Healthcare) pre-equilibrated in gel filtration buffer (50 mM HEPES pH 7.5, 300 mM NaCl, 0.5 mM TCEP). KCTD15 protein fractions were concentrated using centrifugal ultrafiltration and protein concentrations were determined by ultraviolet (UV) absorbance at 280 nm. Sample purity and identity were verified by sodium dodecyl-sulfate polyacrylamide gel electrophoresis (SDS-PAGE) and liquid chromatography–mass spectrometry (MS). Protein was used fresh or flash frozen in liquid nitrogen and stored at −80°C. Attempts to produce the full-length recombinant KCTD15 (residues 1–283) for TFAP2 interaction studies were unsuccessful due to expression in bacterial inclusion bodies. For these assays, we used recombinant KCTD15 BTB+CTD constructs comprising residues 38–283 using the methods above.

### Crystallisation and structure determination

Human KCTD15 BTB domain variant Gly88Asp was concentrated to 10 mg/mL in gel filtration buffer. Crystals were grown at 4°C by sitting-drop vapour-diffusion in 300 nL drops. Crystal form I was obtained by mixing protein in a 2:1 ratio with a reservoir condition containing 17.5% PEG3350, 0.3 M potassium thiocyanate. Crystal form II was obtained by mixing protein in a 1:2 ratio with 12.5% PEG3350, 0.2 M potassium thiocyanate. Prior to vitrification in liquid nitrogen, crystals were cryoprotected in reservoir solution supplemented with 25% ethylene glycol. Diffraction data were collected at the Diamond Light Source beamline I03 then processed in Xia2,^24^ DIALS^25^ or STARANISO.^26^ For crystal form II, molecular replacement was performed using MrBUMP.^27^ Despite an indexing solution indicative of higher symmetry (a≈b, α≈90°, γ≈60°), the *P*1 space group was identified and no higher symmetry solution could be found on reprocessing in DIALS. The solution was then used to phase crystal form I in Phaser.^28^ COOT^29^ was used for manual model building and refinement, whereas PHENIX.REFINE^30^ was used for automated refinement. Structure factors listed in online supplemental table S2 and coordinates were deposited in the PDB with accession codes 8PNR^31^ (crystal form I) and 8PNM^32^ (crystal form II).

### Homology modelling

Homology models of human KCTD15 BTB domain or full-length protein were prepared in the ICM-Pro (Molsoft) software package using KCTD1 PDB codes 5BXB and 6S4L, respectively, as well as for the Asp104His mutant.

### SEC/multiangle light scattering

SEC was performed on an analytical Superose 6 10/300 GL column (GE Healthcare) equilibrated with PBS at a flow rate of 0.5 mL/min. Elution was monitored via online light scattering (DAWN-HELEOS 8+, Wyatt Technology), differential refractive index (Optilab T-rEX, Wyatt Technology) and UV (SPD-20A, Shimadzu) detectors. Proteins were prepared at 1 mg/mL in buffer containing gel filtration buffer and experiments were performed at a flow rate of 0.5 mL/min. Data were analysed using the ASTRA software package v6 (Wyatt Technology).

### Native MS

Proteins were desalted and exchanged into a volatile buffer as described previously^13^ using Micro BioSpin 6 size exclusion columns (Bio-Rad) following the manufacturer’s protocol. In brief, 100 µL of samples at 1 mg/mL were applied to Micro BioSpin 6 size exclusion columns equilibrated with 50 mM ammonium acetate pH 6.5. The eluent was collected and subjected to two additional rounds of size exclusion then held on ice until MS analysis. MS was performed using an Agilent 6530 Quadrupole-Time of Flight (QTOF) mass spectrometer and the following acquisition parameters: ion mode positive; detector mode 1 GHz; scan range 100–20 000 m/z; collision cell off; capillary 3500 V; fragmentor 430 V; skimmer 65 V; octopole rf 750 V; drying gas 325°C; drying gas 5 L/min; nebuliser 15 psi. The instrument was configured using a standard ESI source. The samples were transferred to a 500 µL gas-tight syringe and directly infused into the mass spectrometer at 6 µL/min. Multimer assignment was achieved using an ion table. The complex charge radius was calculated using the method of Testa *et al*.^33^

### Analytical ultracentrifugation

Sedimentation velocity experiments were performed with a Beckman An-60 Ti rotor in a Beckman Optima XL-1 analytical ultracentrifuge at 20°C with a rotor speed of 40 000 rpm. Proteins were prepared at 1 mg/mL in buffer containing 50 mM HEPES pH 7.5, 300 mM NaCl and 0.5 mM TCEP. Radial absorbance scans were recorded using absorbance optics at 280 nm in continuous scan mode. Scans were fitted to a continuous size distribution using SEDFIT,^34^ buffer density and viscosity as well as sample partial specific volumes from amino acid sequences were calculated with SEDNTERP.^35^

### Differential scanning fluorimetry

Protein samples were diluted to 5 µM in gel filtration buffer with 5× SYPRO Orange fluorescent dye. To assess protein thermostability, samples were heated from 25°C to 95°C in an Mx3005P real-time PCR instrument (Stratagene) and fluorescence monitored with excitation and emission filters set to 465 and 590 nm, respectively. Fluorescence data were exported to GraphPad Prism and fitted to the Boltzmann equation to calculate apparent *T*_m_ values as described previously.^36^ All experiments were performed in triplicate.

### Biolayer interferometry

Binding between TFAP2A peptide^37^ and KCTD15 variants was analysed by biolayer interferometry (BLI) performed on an Octet RED384 instrument (FortéBio). Experiments were performed in a buffer containing 25 mM HEPES pH 7.5, 100 mM NaCl and 0.05% (v/v) Tween 20. Biotinylated TFAP2A (AP-2α) peptide (sequence: Biotin-NADFQPPYFPPPYQ, Uniprot P05549-1 residues 50–63) was immobilised onto three streptavidin-coated fibre optic sensor tips (one per KCTD15 variant). A further three ‘free’ reference sensors were used without immobilised peptide. Serial dilutions of the test KCTD15 protein from 10 µM to 100 nM were placed in the relevant wells, with matching buffer in the reference wells. Sensor tips were sequentially dipped into their respective protein samples from low to high concentrations for a 240 s association phase and then into buffer for a 240 s dissociation phase. Data were analysed using the FortéBio Data Analysis software (V.9.0).

## Results

### Identification of heterozygous variants in the BTB domain of KCTD15 in two families affected by complex frontonasal malformation

The clinical study was initiated when we investigated Family 1, in which a father (II-1) and his daughter (III-1) were both affected by a similar combination of frontonasal lipoma, scalp defects, anosmia and congenital heart anomalies (figure 1E, table 1; see online supplemental material for detailed case reports). Trio-based exome sequencing of the affected father and his unaffected parents (I-1) and (I-2) revealed a single high-confidence de novo mutation within the coding portion of the exome, c.310G>C in *KCTD15* (NM_001129994.2, ENST00000683859.1), encoding a p.(Asp104His) substitution within the BTB domain (figure 1B,D,F). No non-synonymous variants at the Asp104 residue are recorded in either the gnomAD (v2.1.1/v3.1.2) (https://gnomad.broadinstitute.org/) or UK Biobank (https://app.genebass.org/) databases of normal allelic variation (combined total of ~986 000 alleles).^38 39^ The c.310G>C variant was shown to segregate to the affected daughter III-1 but not to either of the two unaffected siblings III-2 and III-3 (figure 1E, online supplemental figure 1).

**Table 1:** Summary of clinical features in three individuals heterozygous for *KCTD15* missense variants

	Family 1, II-1	Family 1, III-1	Family 2, II-1
Sex	Male	Female	Female
Histological classification of frontonasal mass	Macroscopically lipoma	Lipoma	Hamartoma
Hypertelorism	Present	Present	Present
Nose	Small nasal tip, short columella	Nasal tip encroached by lipoma	Small nasal tip, short columella
Anosmia	Present	Present	Normal sense of smell
Scalp	Thin hair, balding	Cutis aplasia	Thin, fair hair
Nipples	Normal	Normal	Normal
Congenital heart disease	Tetralogy of Fallot	Patent ductus arteriosus	Not present
Other	Nystagmus, congenital urethral stricture, unilateral renal scarring, dry shiny skin over hands; mild shortening 4th and 5th digits of toes	Tongue-tie, normal extremities	Convergent strabismus, unilateral hearing loss, coeliac disease, 2–3 toe syndactyly

The combination of craniofacial, scalp and cardiac phenotypes in the father and daughter appeared highly distinctive. In particular, although many clinical disorders have been described that include frontonasal malformations, dermoids or lipomas,^40–42^ presentation of frontonasal lipomas with these additional phenotypes appears extremely rare. We found a potential match to a case report describing aplasia cutis congenita, congenital heart lesions and frontonasal cysts in four successive generations,^43^ however, it was not possible to contact the affected family to undertake genetic testing. DNA sequencing of the complete open reading frame of *KCTD15* in 27 unrelated individuals with miscellaneous presentations of frontonasal malformation did not identify any rare non-synonymous heterozygous variants. Intriguingly, however, heterozygous missense variants in the BTB domain of *KCTD1*, which encodes the closest human paralogue of *KCTD15*, cause SEN syndrome, which exhibits a partially overlapping phenotype including cutis aplasia of the scalp.^19 20^ This provided additional circumstantial support that the *KCTD15* variant could be pathogenic in Family 1.

We subsequently investigated Family 2, in which the sporadically affected proband II-1 presented at birth with a midline frontonasal mass (figure 1F). This was surgically removed at the age of 2–4 years; histology of the excised tissue was suggestive of a hamartoma. On review in mid-teens, she was noted to have thin fair hair. Given the clinical similarities to Family 1 we undertook targeted dideoxy-sequencing of *KCTD15*. This demonstrated a heterozygous c.263G>A variant encoding p.(Gly88Asp) in II-1 that was absent in both parental samples, indicating that it had arisen as a de novo mutation (figure 1F, online supplemental figure 1); no non-synonymous variants at the Gly88 residue are recorded in either the gnomAD v2.1.1/v.3.1.2 or UK Biobank databases of normal allelic variation (>986 000 alleles).^38 39^ Of note, the Gly88 residue in the KCTD15 BTB domain occurs at the corresponding position to Gly62 in KCTD1 (figure 1D). Interestingly, an equivalent heterozygous p.(Gly62Asp) substitution at this position in the BTB domain of KCTD1 was previously reported to cause SEN syndrome (figure 1D).^19^

The occurrence of two de novo missense mutations, absent from databases of normal variation and affecting the same domain of the KCTD15 protein, in association with a clinically similar, extremely rare phenotype including frontonasal mass and cutis aplasia or sparse hair, is highly suggestive that these variants are causative of the phenotype. We undertook additional structural and functional analyses to investigate the underlying molecular mechanism.

### Homology modelling predicts that the p.(Asp104His) substitution in Family 1 will disrupt BTB domain oligomerisation

**Figure 2:**
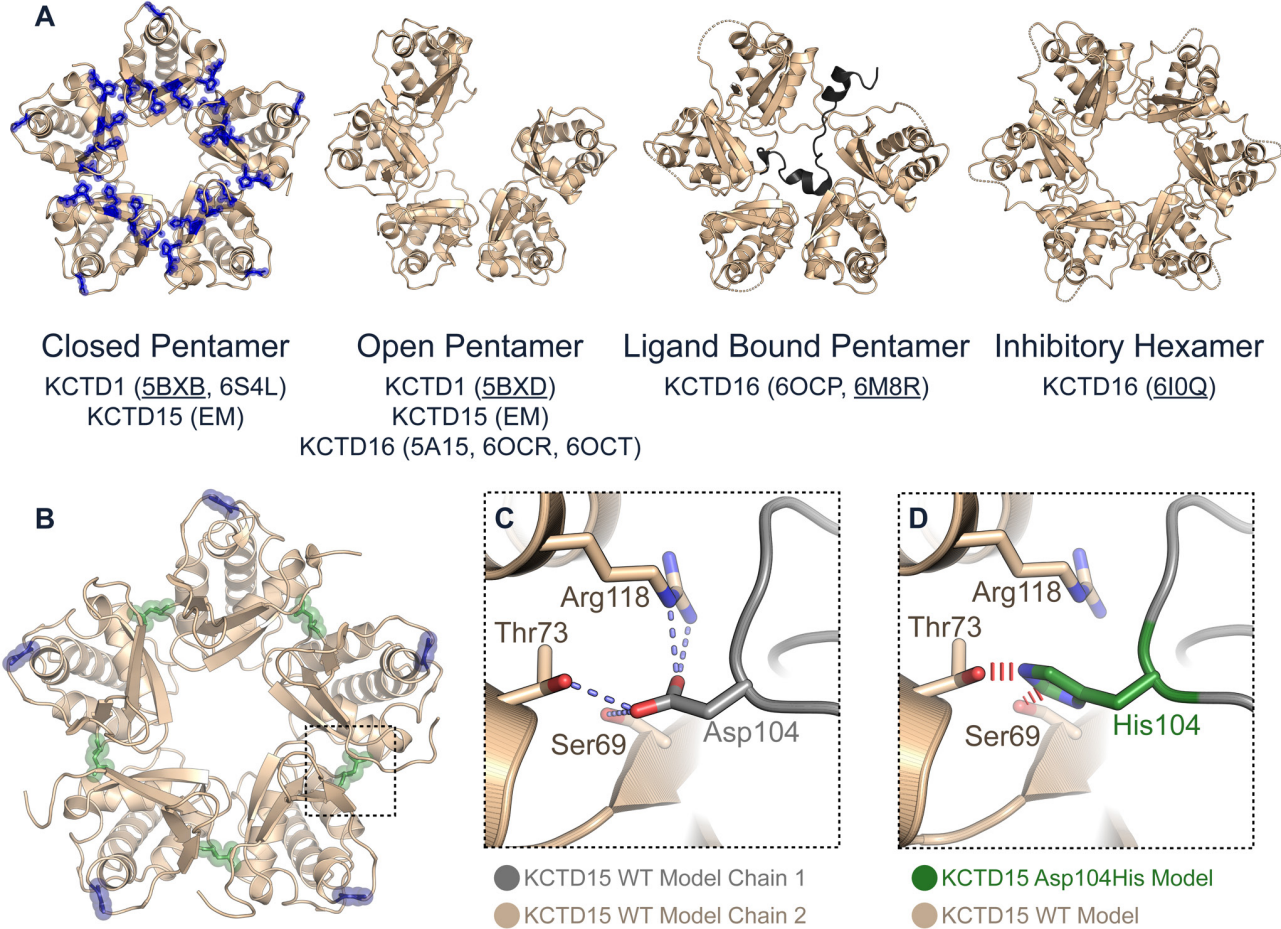
Homology modelling predicts that the Asp104His substitution disrupts BTB domain oligomerisation. (A) Previously determined structural assemblies of the BTB domains of KCTD1, KCTD15 and KCTD16; crystal structure PDB codes are given in parenthesis (PDB used for figure panel is underlined), while electron microscopy (EM) denotes negative stain electron microscopy imaging. KCTD1 and KCTD15 can form open and closed pentameric rings. Blue sticks in closed pentamer model of KCTD1 indicate residues with substitutions in scalp-ear-nipple (SEN) syndrome. KCTD16 adopts a similar open pentameric ring to bind to a GABA_B2_-derived peptide, but this is inhibited if a sixth BTB domain is incorporated into the complex. (B) Homology model of wild-type KCTD15 BTB domain shown as a closed pentamer with the Gly88 and Asp104 side chain positions highlighted in blue and green, respectively. (C) Model of the wild-type KCTD15 BTB domain oligomerisation interface showing the critical intersubunit interactions mediated by Asp104 of one BTB subunit (grey) with Arg118, Ser69 and Thr73 of the neighbouring BTB subunit (brown). (D) Model depicting how the Asp104His substitution (green) will abrogate oligomerisation by breaking the intersubunit salt bridge with Arg118 and introducing steric clashes (red lines) with Ser69 and Thr73.

The atomic resolution structure of KCTD15 has not previously been experimentally determined. However, BTB domain crystal structures have been solved for the close paralogues KCTD1 and KCTD16, demonstrating their assembly into open and closed pentameric rings (figure 2A).^12 13^ The open ring assembly of KCTD16 was shown to function in GABA_B_ receptor signalling by binding to a GABA_B2_-derived peptide,^14 15^ or alternatively to accommodate a sixth BTB subunit to form an inhibitory hexamer (figure 2A).^44^ Similar open and closed pentamers have been observed at low resolution for the BTB domain of KCTD15 using negative stain electron microscopy.^45^ To understand the structural consequences of missense substitutions, we built homology models of the wild-type and mutant BTB domains of KCTD15 using the available structures of KCTD1. Similar to some SEN-causing KCTD1 substitutions (figure 2A), the Asp104His substitution in KCTD15 mapped to the oligomerisation interface (figure 2B) where the wild-type aspartate side chain was predicted to form critical intersubunit interactions, including a salt bridge to Arg118 and hydrogen bonds to Ser69 and Thr73 from the neighbouring BTB subunit (figure 2C). The p.(Asp104His) substitution is predicted to ablate these interactions and inhibit the oligomeric packing relevant for KCTD family interactions.

### Crystal structure of the Gly88Asp mutant BTB domain of KCTD15 (Family 2) shows a closed hexameric assembly

**Figure 3:**
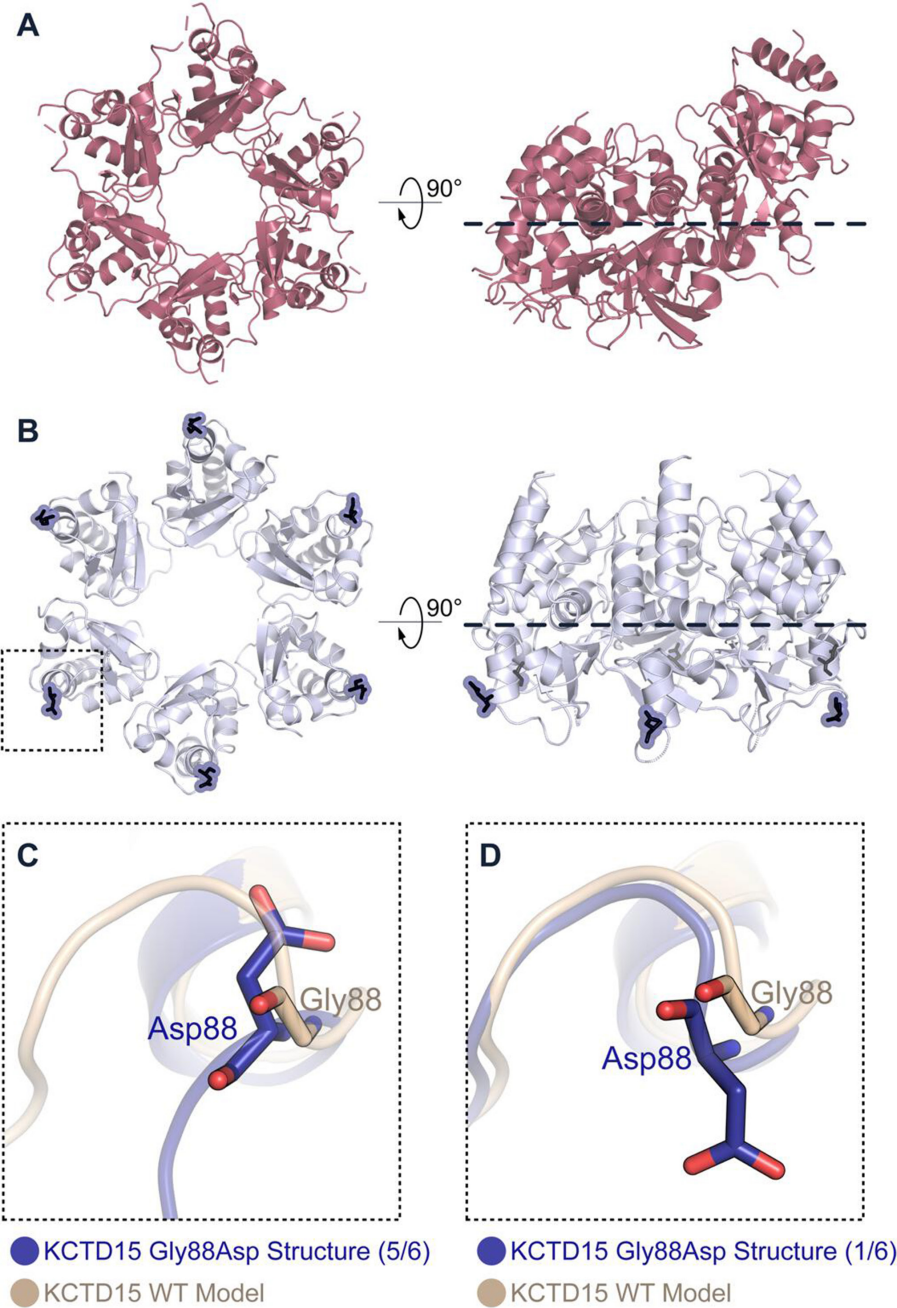
Crystal structure of the Gly88Asp mutant BTB domain of KCTD15 shows a closed hexameric assembly. (A) The previously determined crystal structure of the inhibitory hexamer of KCTD16 is shown in two orientations. A dashed line highlights that the sixth BTB domain subunit is out of plane. (B) Crystal structure of the KCTD15 Gly88Asp mutant BTB domain shown in two orientations (showing crystal form I). The mutant Asp88 residue is shown in each subunit in dark blue. (C,D) Stick representations of the wild-type KCTD15 model (light brown) superimposed on the mutant crystal structure (blue) at the Gly88Asp substitution site. (C) In five of the six BTB subunits in each hexamer the Gly88Asp substitution induced a 180° flip in the main chain relative to the predicted wild-type model (light brown) and displaced the α2-β3 loop by 9 Å. (D) In the remaining BTB subunit the main chain was unaltered by the substitution.

Attempts to purify and crystallise KCTD15 BTB or BTB+CTD protein constructs for structure determination yielded diffracting crystals for the Gly88Asp mutant BTB domain, but not for other wild-type or mutant constructs (online supplemental figures 2 and 3). Two distinct crystal morphologies were obtained, yielding X-ray diffraction datasets for two independent crystal structures solved in space groups *P*4_3_2_1_2 and *P*1 at 2.25 and 1.96 Å resolution, respectively (online supplemental figure 2; see online supplemental table S1 for diffraction data collection and refinement statistics). To our surprise, the Gly88Asp mutant was observed to form a closed hexameric ring in both structures, reminiscent of the inhibitory hexamer of KCTD16 (figure 3A,B); of note, the ring was completely planar in contrast to the KCTD16 hexamer, in which the sixth subunit was a poor fit and out of plane, suggestive of a more stable packing arrangement for the KCTD15 mutant.

The p.(Gly88Asp) substitution (Family 2) occurs in the α2-β3 loop of the BTB domain and is positioned at the solvent-exposed surface of the protein rather than at the oligomerisation interface (figure 3B). As described above, an equivalent SEN-causing p.(Gly62Asp) substitution has been reported at the same position in the BTB domain of KCTD1.^19^ In the absence of a side chain, glycine residues can adopt conformations that are disfavoured for other amino acids. It was noted previously that Gly62 of KCTD1 adopts such a privileged conformation (ϕ, ψ~95°, 0°) and predicted that any substitution would alter this conformation to subtly change the protein structure.^20^ The new structures of the KCTD15 Gly88Asp mutant BTB domain support this prediction. Two hexamers were observed in the asymmetric unit of each KCTD15 structure such that 24 BTB domains (four hexamers) were modelled in total. The main chain at the Gly88Asp substitution site was flipped 180° relative to the wild-type model (and KCTD1 structure) in five of the six BTB domains in each hexamer (figure 3C). As a consequence, the α2-β3 loop, which extends towards the oligomerisation interface, was displaced by 9 Å, suggesting that long-range effects induced the hexameric mutant assembly (figure 3C). The Gly88Asp position in the sixth BTB domain of each hexamer was either disordered or adopted the predicted wild-type conformation (figure 3D).

### Biophysical analyses confirm the perturbed oligomerisation of KCTD15 BTB mutants

**Figure 4:**
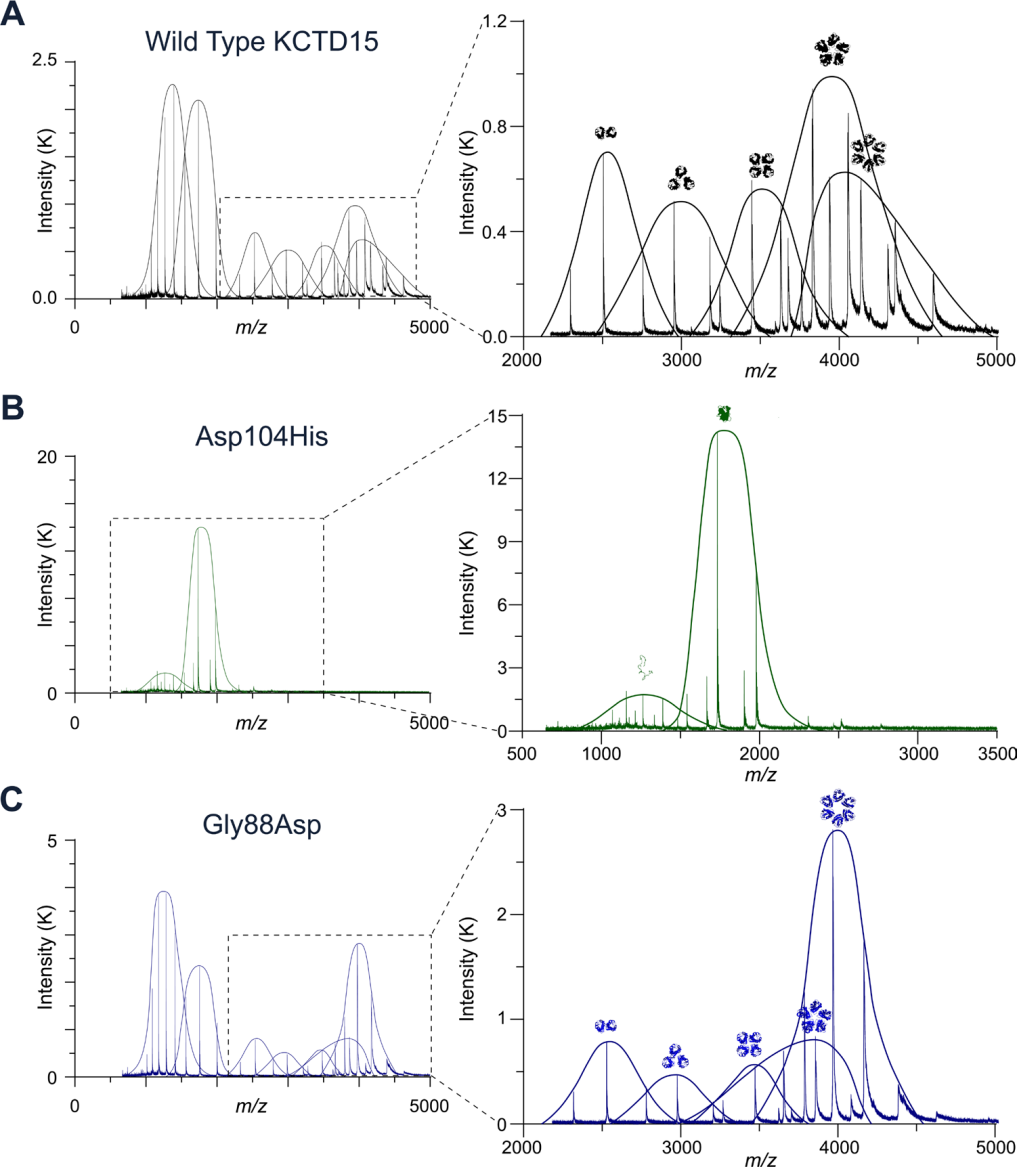
Native mass spectrometry shows perturbed oligomerisation of KCTD15 mutant BTB domains. (A–C) Full native mass spectra of KCTD15 wild-type and mutant BTB domains (left panels) with inset panels (right) highlighting the major species (note peak intensities decrease exponentially as a function of detector efficiency with increasing m/z; the first two peaks of lowest m/z representing unfolded and folded monomers are therefore disproportionately large compared with their populations). A schematic representation of the oligomer state is shown above each peak. (A) Wild-type KCTD15 BTB domain shows a mix of oligomeric species with pentamers predominating (the symmetrical charge distribution of this species, full width half maximum (FWHM) 2.5 charge units, indicates a closed pentamer). (B) KCTD15 BTB mutant Asp104His fails to oligomerise and adopts an exclusively monomeric state. (C) KCTD15 BTB mutant Gly88Asp forms a predominantly hexameric species (also, the lower abundance pentameric species, FWHM 3.0 charge units, shows an asymmetric charge distribution indicating an open flexible structure).

To further validate the structural changes imparted by KCTD15 substitutions, a combination of native MS, SEC coupled with multiangle light scattering (MALS) and analytical ultracentrifugation (AUC) techniques was employed. The electrospray native MS signatures of the BTB domains confirmed that pentamers were the predominant species among a mixture of oligomeric states for wild-type KCTD15 (figure 4A), consistent with previous reports and modelling.^10 45 46^ By contrast, the native MS signature of the Asp104His mutant showed an exclusively monomeric species (figure 4B), providing evidence for our hypothesis that this substitution ablates a salt bridge important for BTB domain oligomerisation. Finally, the Gly88Asp mutant formed predominantly hexameric species consistent with our crystal structure of the same protein. These data show that both KCTD15 substitutions perturb the BTB domain oligomeric state, further supporting that these variants are causative of the phenotype.

The SEC-MALS and AUC analyses also confirmed that the Asp104His mutant BTB protein was monomeric, whereas the Gly88Asp mutant was larger than wild type (online supplemental figure 4). AlphaFold and paralogous structures predict that the CTD of KCTD15 should also assemble into closed pentamers.^10 14 46^ Therefore, the SEC-MALS and AUC studies were additionally performed with constructs containing both the BTB and CTD (herein KCTD15^BTB+CTD^). These proteins exhibited the same trends, but the differences were less pronounced, indicating that oligomerisation of the CTD contributed to their more comparable sizes (online supplemental figure 4).

### The KCTD15 Asp104His mutant is predicted to be partially unfolded at physiological temperatures

To assess how the substitutions and perturbed oligomerisation impacted thermal stability we determined the apparent melting temperatures (*T*_m_) of the different protein variants. Remarkably, the KCTD15^BTB+CTD^ Asp104His mutant displayed an apparent *T*_m_ value of only 35°C, indicating that the Asp104His mutant would be partially unfolded at physiological temperatures (online supplemental figure 4). Moreover, the Asp104His substitution was more destabilising in this multidomain protein than in the isolated KCTD15^BTB^ construct (apparent *T*_m_=49°C), demonstrating that the loss of oligomerisation in one domain affects the stability of the other. By contrast, the Gly88Asp mutant was only destabilised by 1°C relative to wild type with both the KCTD15^BTB^ and KCTD15^BTB+CTD^ constructs exhibiting melting temperatures of 60°C or more (online supplemental figure 4).

### KCTD15 BTB mutants show diminished binding to TFAP2

**Figure 5:**
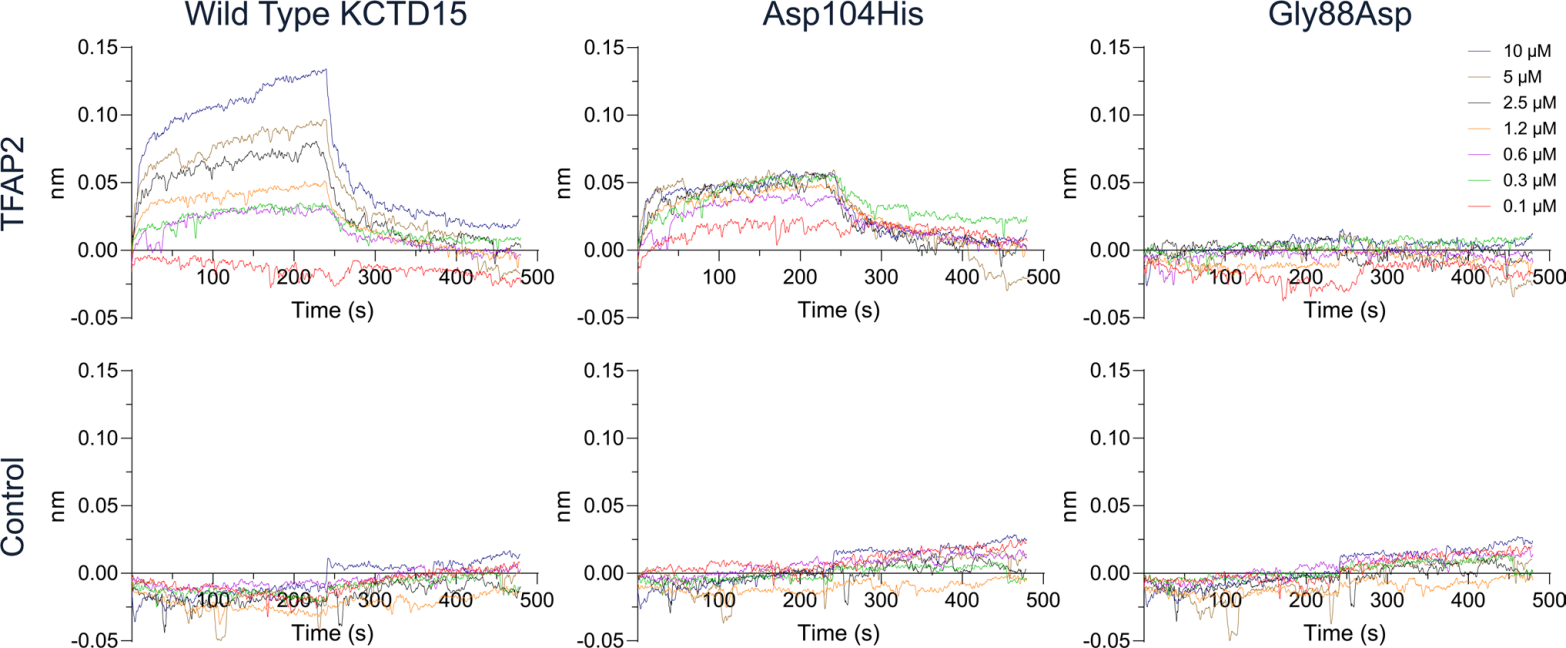
Biolayer interferometry (BLI) measurements show variants Asp104His and Gly88Asp disrupt KCTD15 binding to a TFAP2A peptide. Biotinylated TFAP2A peptide (residues 50–63) was immobilised on streptavidin-coated sensor tips. Tips were incubated in serial dilutions of the indicated KCTD15 variants (residues 38–283) for 240 s to measure their association and then into buffer for 240 s to measure their dissociation (top panel). No binding was observed to streptavidin-coated sensor tips lacking TFAP2A peptide (control lower panel).

Finally, the functional consequences of the Gly88Asp and Asp104His mutations were investigated using BLI to measure the interaction between different KCTD15 variants (BTB+CTD residues 38–283) and a biotinylated TFAP2A peptide captured on streptavidin-coated sensor tips. Wild-type KCTD15 displayed a robust dose–response with progressive increases in TFAP2A binding observed at KCTD15 concentrations up to 10 µM (figure 5). By contrast, the Asp104His mutant showed greatly diminished binding, while the Gly88Asp mutant appeared to have lost binding completely, demonstrating an impaired function for both mutants (figure 5).

The combined structural and biophysical analyses show that the normal pentameric folding and function of the KCTD family proteins is disrupted in KCTD15 mutants, likely resulting in either a partially unfolded monomer for the BTB domain mutant Asp104His, or propensity for a higher order assembly for the Gly88Asp mutant that resembles the inhibitory hexamer of the paralogue KCTD16, suggesting both variants are causal for this novel frontonasal dysplasia syndrome.

## Discussion

Based on observations of three affected individuals from two unrelated families, we present evidence that two amino acid substitutions located within the BTB domain of KCTD15, p.(Gly88Asp) and p.(Asp104His), cause a unique syndrome characterised by a midline mass that overlies the frontonasal region and contains fatty or hamartomatous tissue. Additional clinical features have included sparse scalp hair, aplasia cutis of the scalp and, in Family 1, congenital heart disease (table 1). A similar presentation described as a ‘midline frontal cyst’, accompanied by cutis aplasia of the scalp and congenital heart disease, was reported in a four-generation family in 2007,^43^ however, it has not been possible to establish contact to test whether a *KCTD15* variant is present, and we did not find other convincing phenotypic matches in the literature. While it is important to note that frontonasal malformations have a diversity of causes and presentations,^40 41^ these are often associated with underlying defects of the skull and/or brain; by contrast, there was no evidence for significant brain involvement in any of the three individuals with KCTD15 substitutions whom we identified, although a defect in the anterior cranial fossa was present in the proband from Family 2. Both the p.(Gly88Asp) and p.(Asp104His) variants arose as de novo mutations affecting codons of *KCTD15* that exhibit no non-synonymous variation in nearly 1 million alleles. Although these unique features, together with structural and biophysical evidence that both substitutions perturb higher order assembly of KCTD15, provide strong evidence for a causal link, the identification of additional patients with KCTD15-associated frontonasal dysplasia will clearly be required to delineate the full clinical and molecular spectrum of this disorder. Interestingly, in genome-wide association studies of facial shape, SNPs located near to KCTD15 were reported to affect the morphology of the nasal tip.^47 48^

To explore the mechanisms of pathogenesis we combined biophysical methods (AUC, SEC, MS, BLI) with crystallography to compare the BTB domains of mutant and wild-type KCTD15 proteins. Importantly, KCTD15 normally associates into a multimeric complex, with a pentamer being the most favoured assembly (figure 2A and ref 10 45). We found that both amino acid substitutions disrupted this normal assembly, but interestingly this occurred by different primary mechanisms, which appeared related to the different positions of the amino acids within the KCTD15 monomer. Based both on previous structures and KCTD15 model building, the Asp104 residue is shown to participate in key interactions with the adjacent KCTD15 subunit in the pentamer (figure 2B,C). Substitution of aspartate to histidine is expected to disrupt these interactions (figure 2D) and correspondingly, we found that the Asp104His substitution completely abrogates the formation of BTB domain multimers, but this effect is substantially diminished in the BTB+CTD constructs because of independent multimerisation mediated by the CTD (online supplemental figure 4). By contrast, the Gly88Asp substitution lies on the surface, away from the interdomain interaction region. Substitution of the constrained glycine residue is predicted to alter the local structure; this was confirmed through crystallography which showed that, while higher order assembly of mutant KCTD15 was maintained, a hexameric rather than pentameric state was favoured, with five of six mutant subunits adopting a flipped main chain orientation at the site of the substitution (figure 3). Importantly, although acting by structurally different mechanisms, both substitutions are anticipated to exert a dominant negative effect^49^ by disrupting the assembly of pentamers (a similar mechanism was previously proposed to explain the effects of KCTD1 pathogenic variants in SEN syndrome^19 20^). Only 1 in 32 (calculated as ½^5^) randomly assembling pentamers would consist exclusively of wild-type subunits, hence the consequence of this misassembly is expected to be severe. Interestingly and by contrast, the probability of loss-of-function intolerance (pLI) score of *KCTD15* is 0.04 and the loss of function observed/expected upper bound fraction (LOEUF)^38^ is 0.88, suggesting that the phenotype associated with heterozygous loss-of-function variants in *KCTD15* is likely to be milder or even normal.

How could such proposed abrogation of KCTD15 function lead to the major phenotypic features evident in these patients? In zebrafish, which contains two homologous genes (*kctd15a*, *kctd15b*), expression of both homologues has been documented at the neural plate border immediately lateral to the forming neural crest domain. Later *kctd15a* expression occurs in the pronephric ducts, cranial placode precursors and brain, and *kctd15b* in olfactory placode, cranial neural crest, lateral line primordium, pharyngeal arches, fin buds and optic tectum.^3^ Of particular note, KCTD15 has been documented to be an inhibitor of neural crest development, in part through inhibition of canonical Wnt signalling^3^ and negative regulation of the key neural crest transcription factor TFAP2.^4^ In zebrafish embryos, double knockout of both paralogues (*kctd15a* and *kctd15b*) resulted in expansion of the neural crest domain, manifested as increased expression of neural crest markers and increased pigmentation; in adults, relatively mild maxillary deficiency and absence of facial barbels were also evident.^50^ We speculate that the most characteristic feature of the KCTD15-related phenotype in the three affected individuals, the superficial frontonasal mass, may arise from excess poorly differentiated tissue of neural crest origin, owing to the diminution of normal KCTD15-mediated inhibition. The congenital heart disease in II-1 in Family 1 (tetralogy of Fallot) may also be related to disruption of neural crest. Of interest is that the other prominent feature of the disorder, thinning of hair (especially on the scalp), associated with cutis aplasia in III-1 from Family 1, is also a feature of SEN syndrome caused by pathogenic *KCTD1* variants.^19 20^ Although incompletely understood, it has been proposed that KCTD1-mutant cutis aplasia may reflect excessive TFAP2 transcription factor activity and/or p21 signalling in the skin^51^; it was demonstrated that mutant KCTD1 proteins failed to suppress TFAP2.^37^ The similar presentation of KCTD15 substitutions might reflect shared physiological activity; alternatively, KCTD1 and KCTD15 have been shown to interact^52^ and may form heterodimers, raising the possibility that dominant negative crosstalk between the distinct proteins may contribute to these clinical features. The development of appropriate animal models, and detailed developmental and molecular analysis, will be needed to address the pathological mechanisms definitively.

How could such proposed abrogation of KCTD15 function lead to the major phenotypic features evident in these patients? In zebrafish, which contains two homologous genes (*kctd15a*, *kctd15b*), expression of both homologues has been documented at the neural plate border immediately lateral to the forming neural crest domain. Later *kctd15a* expression occurs in the pronephric ducts, cranial placode precursors and brain, and *kctd15b* in olfactory placode, cranial neural crest, lateral line primordium, pharyngeal arches, fin buds and optic tectum.^3^ Of particular note, KCTD15 has been documented to be an inhibitor of neural crest development, in part through inhibition of canonical Wnt signalling^3^ and negative regulation of the key neural crest transcription factor TFAP2.^4^ In zebrafish embryos, double knockout of both paralogues (*kctd15a* and *kctd15b*) resulted in expansion of the neural crest domain, manifested as increased expression of neural crest markers and increased pigmentation; in adults, relatively mild maxillary deficiency and absence of facial barbels were also evident.^50^ We speculate that the most characteristic feature of the KCTD15-related phenotype in the three affected individuals, the superficial frontonasal mass, may arise from excess poorly differentiated tissue of neural crest origin, owing to the diminution of normal KCTD15-mediated inhibition. The congenital heart disease in II-1 in Family 1 (tetralogy of Fallot) may also be related to disruption of neural crest. Of interest is that the other prominent feature of the disorder, thinning of hair (especially on the scalp), associated with cutis aplasia in III-1 from Family 1, is also a feature of SEN syndrome caused by pathogenic *KCTD1* variants.^19 20^ Although incompletely understood, it has been proposed that KCTD1-mutant cutis aplasia may reflect excessive TFAP2 transcription factor activity and/or p21 signalling in the skin^51^; it was demonstrated that mutant KCTD1 proteins failed to suppress TFAP2.^37^ The similar presentation of KCTD15 substitutions might reflect shared physiological activity; alternatively, KCTD1 and KCTD15 have been shown to interact^52^ and may form heterodimers, raising the possibility that dominant negative crosstalk between the distinct proteins may contribute to these clinical features. The development of appropriate animal models, and detailed developmental and molecular analysis, will be needed to address the pathological mechanisms definitively.

How could such proposed abrogation of KCTD15 function lead to the major phenotypic features evident in these patients? In zebrafish, which contains two homologous genes (*kctd15a*, *kctd15b*), expression of both homologues has been documented at the neural plate border immediately lateral to the forming neural crest domain. Later *kctd15a* expression occurs in the pronephric ducts, cranial placode precursors and brain, and *kctd15b* in olfactory placode, cranial neural crest, lateral line primordium, pharyngeal arches, fin buds and optic tectum.^3^ Of particular note, KCTD15 has been documented to be an inhibitor of neural crest development, in part through inhibition of canonical Wnt signalling^3^ and negative regulation of the key neural crest transcription factor TFAP2.^4^ In zebrafish embryos, double knockout of both paralogues (*kctd15a* and *kctd15b*) resulted in expansion of the neural crest domain, manifested as increased expression of neural crest markers and increased pigmentation; in adults, relatively mild maxillary deficiency and absence of facial barbels were also evident.^50^ We speculate that the most characteristic feature of the KCTD15-related phenotype in the three affected individuals, the superficial frontonasal mass, may arise from excess poorly differentiated tissue of neural crest origin, owing to the diminution of normal KCTD15-mediated inhibition. The congenital heart disease in II-1 in Family 1 (tetralogy of Fallot) may also be related to disruption of neural crest. Of interest is that the other prominent feature of the disorder, thinning of hair (especially on the scalp), associated with cutis aplasia in III-1 from Family 1, is also a feature of SEN syndrome caused by pathogenic *KCTD1* variants.^19 20^ Although incompletely understood, it has been proposed that KCTD1-mutant cutis aplasia may reflect excessive TFAP2 transcription factor activity and/or p21 signalling in the skin^51^; it was demonstrated that mutant KCTD1 proteins failed to suppress TFAP2.^37^ The similar presentation of KCTD15 substitutions might reflect shared physiological activity; alternatively, KCTD1 and KCTD15 have been shown to interact^52^ and may form heterodimers, raising the possibility that dominant negative crosstalk between the distinct proteins may contribute to these clinical features. The development of appropriate animal models, and detailed developmental and molecular analysis, will be needed to address the pathological mechanisms definitively.

How could such proposed abrogation of KCTD15 function lead to the major phenotypic features evident in these patients? In zebrafish, which contains two homologous genes (*kctd15a*, *kctd15b*), expression of both homologues has been documented at the neural plate border immediately lateral to the forming neural crest domain. Later *kctd15a* expression occurs in the pronephric ducts, cranial placode precursors and brain, and *kctd15b* in olfactory placode, cranial neural crest, lateral line primordium, pharyngeal arches, fin buds and optic tectum.^3^ Of particular note, KCTD15 has been documented to be an inhibitor of neural crest development, in part through inhibition of canonical Wnt signalling^3^ and negative regulation of the key neural crest transcription factor TFAP2.^4^ In zebrafish embryos, double knockout of both paralogues (*kctd15a* and *kctd15b*) resulted in expansion of the neural crest domain, manifested as increased expression of neural crest markers and increased pigmentation; in adults, relatively mild maxillary deficiency and absence of facial barbels were also evident.^50^ We speculate that the most characteristic feature of the KCTD15-related phenotype in the three affected individuals, the superficial frontonasal mass, may arise from excess poorly differentiated tissue of neural crest origin, owing to the diminution of normal KCTD15-mediated inhibition. The congenital heart disease in II-1 in Family 1 (tetralogy of Fallot) may also be related to disruption of neural crest. Of interest is that the other prominent feature of the disorder, thinning of hair (especially on the scalp), associated with cutis aplasia in III-1 from Family 1, is also a feature of SEN syndrome caused by pathogenic *KCTD1* variants.^19 20^ Although incompletely understood, it has been proposed that KCTD1-mutant cutis aplasia may reflect excessive TFAP2 transcription factor activity and/or p21 signalling in the skin^51^; it was demonstrated that mutant KCTD1 proteins failed to suppress TFAP2.^37^ The similar presentation of KCTD15 substitutions might reflect shared physiological activity; alternatively, KCTD1 and KCTD15 have been shown to interact^52^ and may form heterodimers, raising the possibility that dominant negative crosstalk between the distinct proteins may contribute to these clinical features. The development of appropriate animal models, and detailed developmental and molecular analysis, will be needed to address the pathological mechanisms definitively.

How could such proposed abrogation of KCTD15 function lead to the major phenotypic features evident in these patients? In zebrafish, which contains two homologous genes (*kctd15a*, *kctd15b*), expression of both homologues has been documented at the neural plate border immediately lateral to the forming neural crest domain. Later *kctd15a* expression occurs in the pronephric ducts, cranial placode precursors and brain, and *kctd15b* in olfactory placode, cranial neural crest, lateral line primordium, pharyngeal arches, fin buds and optic tectum.^3^ Of particular note, KCTD15 has been documented to be an inhibitor of neural crest development, in part through inhibition of canonical Wnt signalling^3^ and negative regulation of the key neural crest transcription factor TFAP2.^4^ In zebrafish embryos, double knockout of both paralogues (*kctd15a* and *kctd15b*) resulted in expansion of the neural crest domain, manifested as increased expression of neural crest markers and increased pigmentation; in adults, relatively mild maxillary deficiency and absence of facial barbels were also evident.^50^ We speculate that the most characteristic feature of the KCTD15-related phenotype in the three affected individuals, the superficial frontonasal mass, may arise from excess poorly differentiated tissue of neural crest origin, owing to the diminution of normal KCTD15-mediated inhibition. The congenital heart disease in II-1 in Family 1 (tetralogy of Fallot) may also be related to disruption of neural crest. Of interest is that the other prominent feature of the disorder, thinning of hair (especially on the scalp), associated with cutis aplasia in III-1 from Family 1, is also a feature of SEN syndrome caused by pathogenic *KCTD1* variants.^19 20^ Although incompletely understood, it has been proposed that KCTD1-mutant cutis aplasia may reflect excessive TFAP2 transcription factor activity and/or p21 signalling in the skin^51^; it was demonstrated that mutant KCTD1 proteins failed to suppress TFAP2.^37^ The similar presentation of KCTD15 substitutions might reflect shared physiological activity; alternatively, KCTD1 and KCTD15 have been shown to interact^52^ and may form heterodimers, raising the possibility that dominant negative crosstalk between the distinct proteins may contribute to these clinical features. The development of appropriate animal models, and detailed developmental and molecular analysis, will be needed to address the pathological mechanisms definitively.

An analysis of the biological consequences of the KCTD15 p.Asp104. His substitution identified in Family 1, based on a meeting abstract presented by our group, was recently published.^53^ This work independently supports a dominant negative mechanism of action as proposed here, in part through disruption of KCTD1/KCTD15 heteropentamers.

## Data Availability

Data are available in a public, open access repository. Structure factors and coordinates were deposited in the PDB with accession codes 8PNR (crystal form I) and 8PNM (crystal form II). All other data relevant to the study are included in the article.
