# Supplementary material for: BTB domain mutations perturbing KCTD15 oligomerisation cause a distinctive frontonasal dysplasia syndrome
Source: J Med Genet. 2024 Jan 31;61(5):490–501. doi: 10.1136/jmg-2023-109531 (PMC11041601; doi:10.1136/jmg-2023-109531)
Supplement: Supplementary data [file jmg-2023-109531supp001.pdf]

BTB domain mutations perturbing KCTD15 oligomerisation cause a distinctive frontonasal dysplasia syndrome

Kerry A. Miller, David A. Cruz Walma *et al.*

SUPPLEMENTARY MATERIAL

Family 1

The female proband, III-1, was born by caesarean section at 42 weeks' gestation. This followed an uncomplicated pregnancy, although routine ultrasound scanning had raised the possibility that the fetus might have a cleft palate. The family history was notable in that the father (II-1) had been born with multiple congenital abnormalities (see below), and the mother (II-2) had a diagnosis of multiple exostoses. Two younger siblings, a sister (III-2) and brother (III-3), were subsequently born, who both have a normal facial appearance.

At birth the proband was noted to have a prominent symmetrical mass extending from the nasal bridge to the forehead suggestive of a lipoma; in addition there was extensive cutis aplasia involving the posterior half of the scalp. Magnetic resonance imaging performed in the neonatal period showed no evidence of clefts or intracerebral extension of the frontonasal lipoma. Nasoendoscopy showed narrowing of the airways, but excluded choanal atresia. By the age of 4.5 months (mo) the area of cutis aplasia was noted to have healed. In addition she had required surgical division of a tongue-tie and was being fed using a nasogastric tube. Echocardiogram demonstrated a moderate-sized patent ductus arteriosus, which was repaired at the age of 5 mo. An ultrasound of the kidneys was normal.

She was first seen for craniofacial assessment at the age of 6 mo. The frontonasal lipoma was noted to be associated with telecanthus and hypertelorism, and appeared to be encroaching on her medial vision. However formal ophthalmological and orthoptic reviews

Miller KA, *et al. J Med Genet* 2024; 61:490–501. doi: 10.1136/jmg-2023-109531

were normal. Her nostrils were small and there was no visible nasal bridge. The ears were mildly low-set but with normal morphology. She continued to be fed by nasogastric tube but ENT assessment revealed a satisfactory nasal airway and a high-arched palate, with no anatomical problem, suggesting a functional cause for this. The remainder of the physical examination was normal, in particular the extremities including digits and nails were noted to be normal.

Computed tomography (CT) scan of the head showed a large area of subcutaneous fat hypertrophy, without a defined border, and dura was visualised deep to the nasal defect. A small bony defect was evident on the vertex of the calvaria. The brain was structurally normal.

The lipoma was surgically excised at the age of 15 mo following a bicoronal incision, with a subgaleal flap raised to the supraorbital rim and continued in the subperiosteal plane to the nasal dorsum. The lipoma was found to be diffuse with no clear plane of separation from the otherwise normal subcutaneous fat. The excess fat was removed, and the marked excess of skin over the nasal dorsum and forehead was excised. She recovered well with good wound healing. A divergent squint noted at this time did not require any active management.

Evaluation of language skills at the time of admission for surgery using the Receptive-Expressive Emergent Language Test – 3rd Edition (REEL-3) gave a standard score of 100 (58% rank) for receptive skills, 73 (3% rank) for expressive skills, and 86 (18% rank) overall, indicating appropriate receptive language skills but moderately-delayed expressive language skills. Neuropsychological evaluation (Bayley Scales of Infant and Toddler Development, 3rd UK edition) gave a composite score of 90 (25% rank) for cognitive development and 67 (1% rank) for motor development. Reflecting the low motor score, she had been late to crawl, and walked independently just before the age of 2 years. Audiological assessment showed a

Miller KA, *et al. J Med Genet* 2024; 61:490–501. doi: 10.1136/jmg-2023-109531

mild conductive hearing loss with middle ear fluid present bilaterally, which later (at the age of 4 years [yr] 8 mo) was managed with grommets.

Review at the age of 2 yr 9 mo revealed mild short stature (height 84.1 cm, 0.4 centile [%]), with normal weight (12.5 kg, 25%). Thyroid function tests and IGF1 were normal. The height continued to track along the 0.4% line. At 4 years she was noted to have low myopia (0.2 LogMAR), and was later prescribed spectacles. The enamel on her teeth was noted to be thin and she had a long upper labial frenum. Nipple development was normal.

Formal community paediatric review was undertaken at 5 yr 2 mo. Her height was 97.9 cm (<0.4%), weight 15.2 kg (25%) and occipito-frontal circumference 48.1 cm (0.4%). She attended a normal school but had specific difficulties with handwriting and balance. Multiple small bony lumps were noted on her ribs, both shoulders and both knees, indicating the presence of multiple exostoses separately inherited from her affected mother.

Formal assessment of her coordination difficulties was undertaken at the age of 6 yr. The Movement ABC-2 assessment showed a normal score for manual dexterity (37%), but very low scores for ball skills and static and dynamic balance (0.1%). The Test of Visual Perceptual Skills yielded mostly normal scores, but with specific difficulties with visual memory (1%) and visual spatial relationships (1%). These results were considered compatible with Developmental Coordination Disorder.

The father II-1 had also been born with cutis aplasia of the scalp and a large facial lipoma, to unaffected non-consanguineous parents (father I-1, mother I-2), who were aged 30 yr and 27 yr respectively at the time of his birth. In addition he required surgery in infancy for tetralogy of Fallot and a hypoplastic urethra. Facial reconstruction surgery was performed at the age of 3 yr: the subcutaneous lipoma was noted to extend into the face across the root of the nose, but not to communicate intracerebrally. The canthal ligaments were shortened and

reattached by transnasal wiring. He had normal development and schooling and works as a nurse. He stated that he had never had a sense of smell and that this feature was also present in III-1. As an adult he developed frequent urinary tract infections and ultrasound of the kidneys showed unilateral renal scarring.

On examination he was noted to have scars on his nose and scalp related to his previous surgery, thin atrophic skin over his scalp, mild hypertelorism with arched eyebrows and a small nasal tip associated with a very short columella. Nystagmus was present. His hands exhibited dry, shiny skin but were otherwise normal; mild shortening of the 4th and 5th toes was present bilaterally. Nipple development was normal.

Initial genetic testing of the proband III-1 included array comparative genomic hybridisation using the 60k v2.0 ISCA array, and targeted sequencing of *ZSWIM6*, which were both normal. Following informed consent, blood was obtained from the unaffected grandparents I-1 and I-2, together with II-1, for trio-based exome sequencing based on the hypothesis that a *de novo* mutation had occurred in II-1 to account for his phenotype and transmission to his daughter III-1. Conditional Bayesian analysis was used to prioritise variants present in the offspring (II-1) that were absent in parental reads. This identified three apparently *de novo* mutations, chr19:33806930G>C (c.310G>C predicted to encode a p.(Asp104His) substitution in *KCTD15,* ENST00000430256.3), chr19:38106457A>T (c.2030-80A>T in *SIPA1L3*, ENST00000222345.11) and chr21:32726684C>T (c.241+88G>A in *SYNJ1*, ENST00000382499.7). The latter two variants fall within introns of the corresponding genes; analysis using the Human Splicing Finder (Genomnis) predicted no impact on splicing. Hence the *KCTD15* variant, located within exon 5, was prioritised for further investigation.

PCR was used to amplify the complete open reading frame of *KCTD15* (exons 3-7). Primers are provided in Table S1. Dideoxy-sequencing and HphI-digestion of the exon 5 PCR product were used to confirm the c.310G>C transversion (supplementary figure 1, left).

Family 2

The proband is a 17 yr old female, the first child born to non-consanguineous parents who were aged 28 yr (father) and 34 yr (mother) at the time of her birth. She has a younger sibling who is well. Her parents are both well. Her father has a paternal cousin born with cleft lip and a maternal cousin born with cleft lip and palate. Her mother has a paternal cousin born with cleft lip. There is no other relevant family history.

There were concerns regarding growth during the pregnancy from 30 weeks' gestation and therefore growth was monitored with regular antenatal scans. Labour was induced at 38 wk, and she was born by caesarean section, with a birth weight of 2.3 kg (2%).

At birth a midline mass in the frontonasal region was apparent, which was associated with nasal hypoplasia and hypertelorism. She had bilateral convergent strabismus which was surgically corrected at the age of 3 years. She had mild unilateral congenital hearing loss in the left ear, which did not require a hearing aid.

Her development was noted to be normal at a clinical genetics review at the age of 2 yr 4 mo. Examination showed hypertelorism with a straight nose and hypoplastic nares. CT imaging at that time did not show any evidence of craniosynostosis, but confirmed a symmetrical midline soft tissue swelling in the frontal region, extending down the root of the nose. There was a persistent metopic suture, but no abnormal widening of this suture. The nasal septum was abnormally wide. This abnormal widening extended to the skull base in the region of the crista galli, which was deficient in its anterior half. Subsequent MRI imaging showed no brain abnormality, a widened nasal septum, and confirmed the abnormality of the crista galli shown on CT, but no soft tissue abnormality was seen in association with this. The facial mass was surgically removed at the age of 3 yr. Histology showed features in keeping with a hamartoma.

Miller KA, *et al. J Med Genet* 2024; 61:490–501. doi: 10.1136/jmg-2023-109531

The proband was referred back to the genetics clinic at the age of 15 yr. She had normal intelligence and attended a mainstream school. She suffered from coeliac disease, managed by diet. She wore spectacles for mild myopia. Her sense of taste and smell was normal. On examination her growth parameters showed a head circumference of 56.0 cm (75%), height 161.5cm (25-50%) and weight 48 kg (25%). She had fair, thin hair. She was hyperteloric with hypoplastic nares. She had mild frontal bossing. She had 2-3 syndactyly of the toes bilaterally. Her thumb nails had longitudinal ridging bilaterally. She had a single hypopigmented patch on the right shoulder. Her cardiac examination was normal. Nipple and breast development were normal. There was no evidence of cutis aplasia. A repeat CT head scan at the age of 15 yr showed multiple irregularities of the inferior frontal bone, but no breach of the inner or outer table. There was no specific skull base abnormality identified. No imaging of the kidneys or lower urinary tract had been performed.

Array CGH showed a variant of unknown significance, a 6 kb gain at 11p15.4 involving the *HBG1* and *HBG2* genes, thought unlikely to be relevant to her phenotype. Initial genetic testing included sequencing of *ALX1*, *ALX3*, *ALX4* and *PTEN*, which were normal. A craniosynostosis gene panel (including sequencing of selected exons of *FGFR1*, *FGFR2*, *FGFR3*, *TWIST1*, *TCF12*, *ERF* and *EFNB1* and multiplex ligation-dependent probe amplification (MLPA) of *TWIST1*, *RUNX2*, *ALX1*, *ALX3*, *ALX4*, *MSX2*, *EFNB1*, *TCF12* and *ERF*) was also normal. Dideoxy-sequencing of *KCTD15* was subsequently undertaken based on the similarity of clinical features to those in Family 1. Confirmation of the c.263G>A transition in II-1 was provided using a reverse sequencing primer (supplementary figure 1, right).

Table S1 PCR amplification of *KCTD15*.

Exon #	Forward primer (5′-3′)	Reverse primer (5′-3′)	Product length (bp)	Tm (°C)
3	GTGGAGTCAATCAGGGAGGG	CAAGGAAGGACAGCCAGTAA	321	58
4	CTAACACATAAGCCCACGACC	TAGCAGCTTTCAACAGGCAG	693	58
5	TCCAGAGTCACCCATGTCAG	ACAACCAGGGGTCCACTTAT	366	58
6	TCCTGCAGAATTGGTTGGAAC	AGACCACCTTTCCACTCAGC	780	58
7	TCAGGGAAGCTGGTGGATG	CCTCACCCTCGGTCTCAT	699	58

Miller KA, *et al. J Med Genet* 2024; 61:490–501. doi: 10.1136/jmg-2023-109531

Table S2 Crystallographic data collection and refinement statistics

	**Crystal form I PDB: 8PNR**	**Crystal form II PDB: 8PNM**
**Data Collection**		
Wavelength	0.976254	0.976254
Resolution range	78.0 - 2.25 (2.29-2.25)	91.3 - 1.95 (2.12 - 1.96)
Space group	$P4_32_12$	$P1$
Unit cell dimensions:		
a, b, c (Å)	151.1, 151.1, 156.0	71.3, 72.1, 94.4
α, β, γ (°)	90.0, 90.0, 90.0	89.6, 77.1, 60.5
Total reflections	2341293	276137 (13890)
Unique reflections	85815 (4227)	75791 (3791)
Multiplicity	27.28	3.6
Completeness (%)	100.0 (100.0)	92.5 (61.2)
Mean I/sigma(I)	14.8	14.2
Wilson B-factor	54.44	37.99
R-merge	0.127 (4.460)	0.044 (0.797)
R-meas	0.130	0.052
R-pim	0.025	0.027
CC1/2	0.9945	0.999
**Refinement**		
R-work	0.246	0.238
R-free	0.285	0.273
Number of non-hydrogen atoms	18138	9618
Macromolecules	18049	9372
Ligands	0	0
Solvent	89	246
Protein residues	1191	1215
RMS(bonds, Å)	0.009	0.005
RMS(angles, °)	1.246	1.090
Ramachandran favored (%)	94.83	95.29
Ramachandran allowed (%)	4.73	4.37
Ramachandran outliers (%)	0.44	0.34
Rotamer outliers (%)	1.56	0.22
Clashscore	7.76	3.9
Average B-factor (Å^2)	79.16	57.79
Macromolecules	81.12	57.93
Ligands	NA	NA
Solvent	65.59	45.50

Statistics for the highest-resolution shell are shown in parentheses.

Miller KA, *et al. J Med Genet* 2024; 61:490–501. doi: 10.1136/jmg-2023-109531

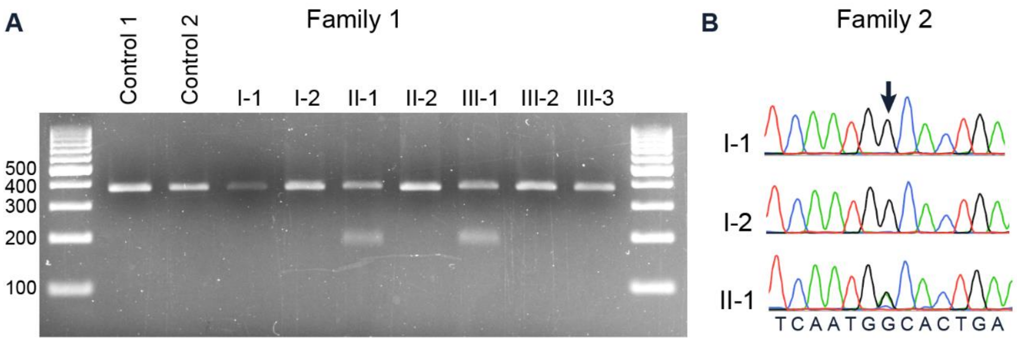

Supplemental figure 1

Additional confirmation of *KCTD15* sequence variants. (A) HphI restriction digest of *KCTD15* exon 5 in Family 1. (B) Chromatograms of *KCTD15* exon 5 sequenced using reverse primer, for individuals in Family 2.

Miller KA, *et al. J Med Genet* 2024; 61:490–501. doi: 10.1136/jmg-2023-109531

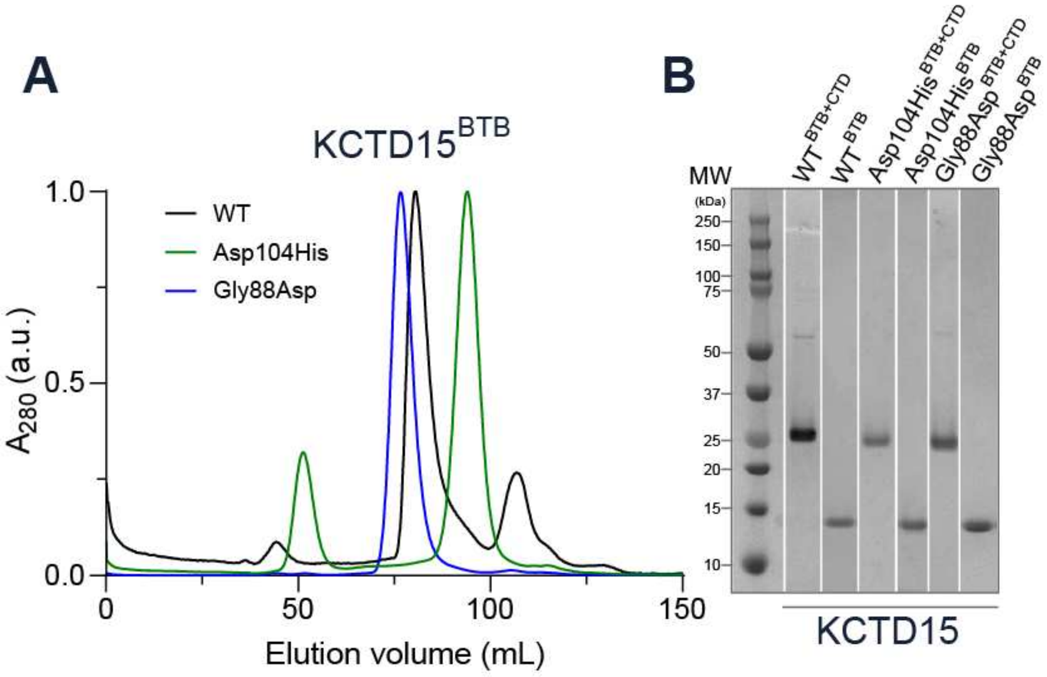

Supplemental figure 2

Purification and crystallisation of KCTD15 proteins. (A) Representative preparative size exclusion chromatography of KCTD15 BTB domain variants, including wild type, Gly88Asp mutant and Asp104His mutant. Size differences are evident consistent with the analytical SEC-MALS experiments. (B) Coomassie-stained SDS PAGE gel of purified proteins.

Miller KA, *et al. J Med Genet* 2024; 61:490–501. doi: 10.1136/jmg-2023-109531

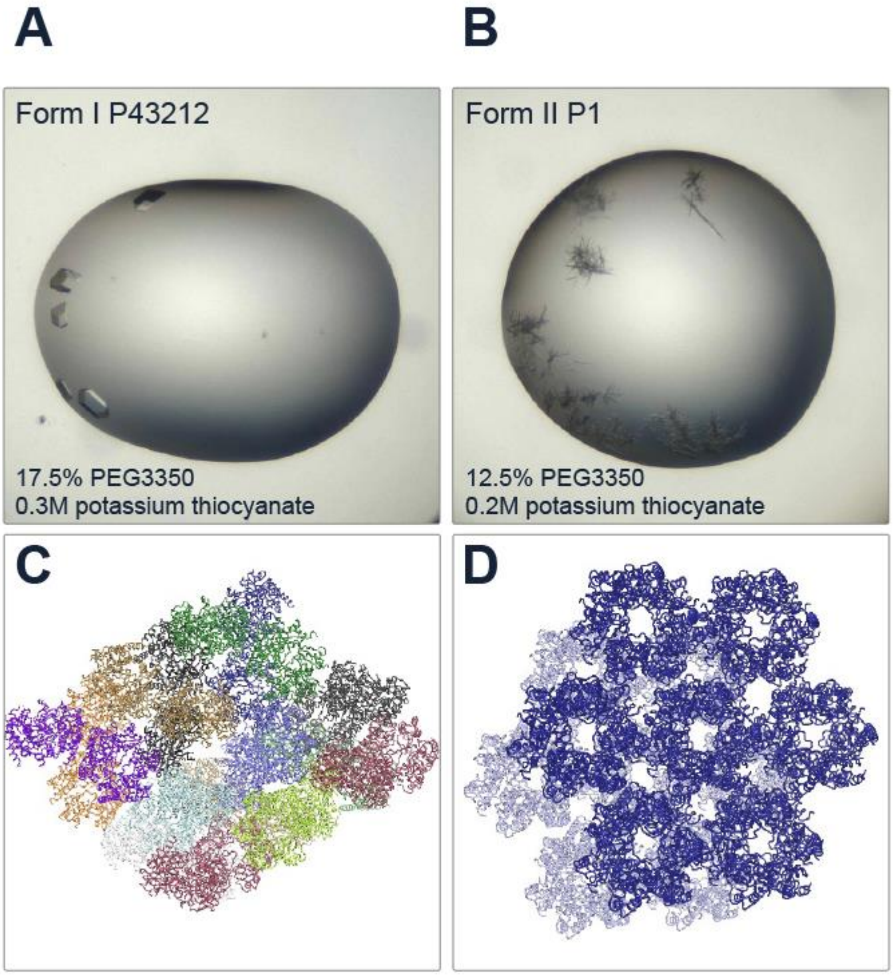

Supplemental figure 3

Two crystal forms for the KCTD15 Gly88Asp BTB domain. Photographic images of KCTD15 Gly88Asp crystal form I (A) and crystal form II (B). Crystal lattice images for KCTD15 Gly88Asp crystal form I (C) and crystal form II (D).

Miller KA, *et al. J Med Genet* 2024; 61:490–501. doi: 10.1136/jmg-2023-109531

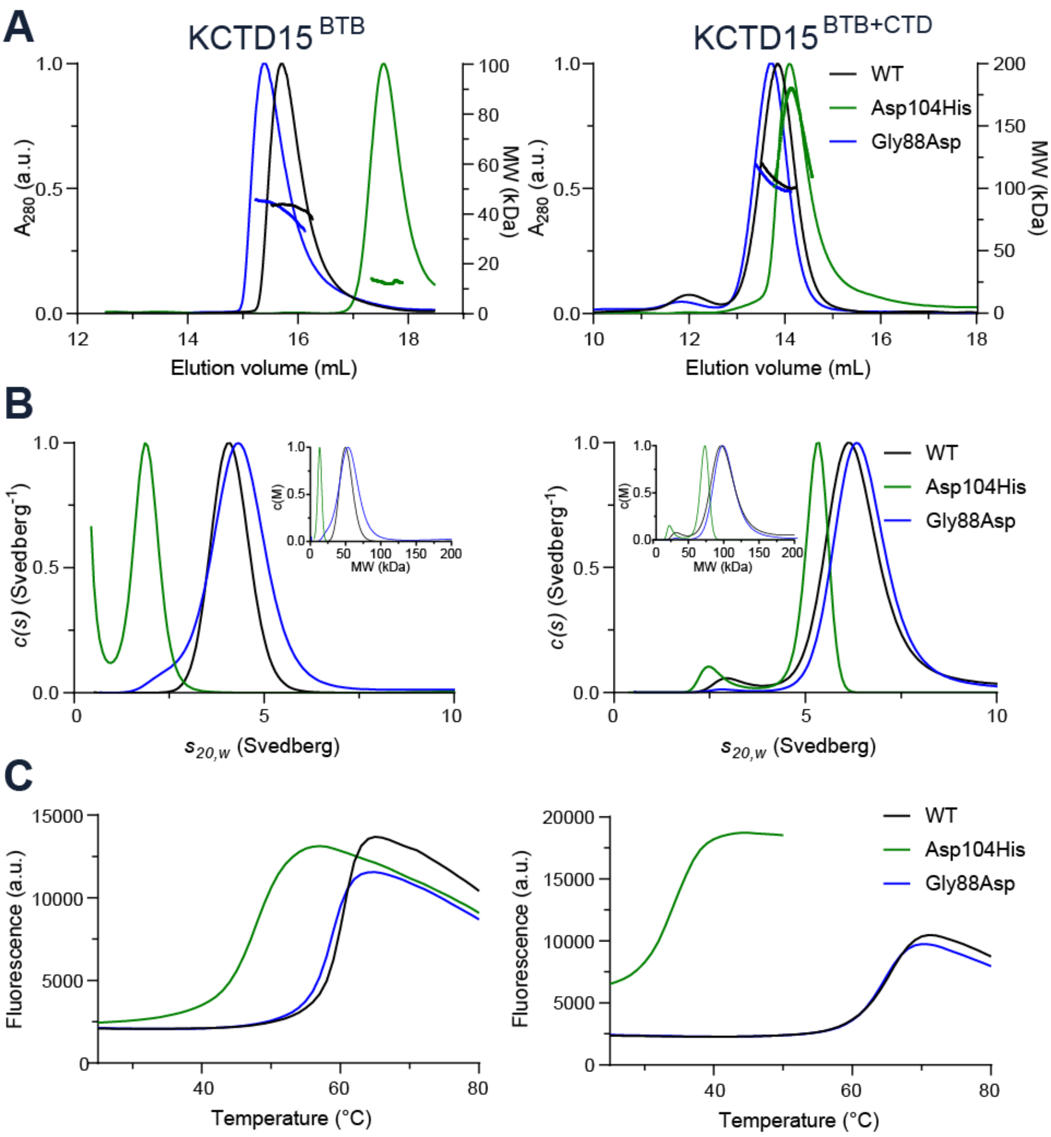

Supplemental figure 4

Biophysical analyses of KCTD15 wild-type and mutant proteins. (A-C) Analyses of KCTD15 BTB domains (KCTD15^{BTB} left panels) and BTB+CTD constructs (KCTD15$^{BTB+CTD}$ right panels). Different KCTD15 variants are coloured black (wild type), green (Asp104His) or blue (Gly88Asp), respectively. (A) SEC-MALS analyses of KCTD15 variants showing the SEC elution absorbance (280 nm, left *y* axis) and MALS-estimated masses (right *y* axis). A monomer was observed for the Asp104His KCTD15^{BTB} construct, whereas the Gly88Asp mutant BTB was larger than wild type. The KCTD15$^{BTB+CTD}$ constructs exhibited the same trend, but the differences were less pronounced, indicating that C-terminal domain oligomerisation contributed to their more comparable sizes. A gradient in the MALS data

suggested that the Gly88Asp mutants, and to a lesser extent wild-type proteins, adopted heterogenous oligomeric states suggesting some dynamic behaviour in solution. Their predicted average assemblies across the MALS experiments ranged from pentamers and tetramers (KCTD15$^{BTB+CTD}$) to trimers (KCTD15^{BTB}) (B) Sedimentation velocity AUC data for KCTD15 variants. Differential sedimentation coefficient distributions [c(s)] are plotted versus apparent sedimentation coefficients (s20,w) (main graphs) as well as differential molecular weight distributions [c(M)] versus molecular weights (MW) (insets). The Asp104His KCTD15^{BTB} mutant was monomeric, whereas the Gly88Asp mutant was again larger than wild type (although both were predicted tetramers). Differences were less pronounced for the KCTD15$^{BTB+CTD}$ constructs. Broadening of the AUC peak for the Gly88Asp KCTD15^{BTB} construct was consistent with the heterogenous oligomeric states suggested by the MALS data. (C) Thermal denaturation of KCTD15 variants detected using the SYPRO Orange fluorescent dye which binds to exposed hydrophobic surfaces upon protein unfolding. For the wild type, the multidomain KCTD15$^{BTB+CTD}$ construct was 5°C more stable than the isolated KCTD15^{BTB} (apparent T_m values of 66 and 61°C, respectively). Remarkably, the KCTD15$^{BTB+CTD}$ Asp104His mutant displayed an apparent T_m value of only 35°C, indicating that the Asp104His mutant would be partially unfolded at physiological temperatures. Moreover, the Asp104His substitution was more destabilising in this multidomain protein than in the isolated KCTD15^{BTB} construct (apparent T_m = 49°C), which was opposite to the wild-type protein. This suggests that the loss of oligomerisation in one domain affects the stability of the other. The decreased thermostability of the Asp104His KCTD15^{BTB} construct (apparent T_m = 49°C) compared to the wild-type KCTD15^{BTB} (apparent T_m = 61°C) is likely entirely due to the lost intersubunit interactions of the wild-type pentamer. Finally, for the Gly88Asp mutant, both the KCTD15$^{BTB+CTD}$ and KCTD15^{BTB} constructs were destabilised by 1°C relative to wild type.

Miller KA, *et al. J Med Genet* 2024; 61:490–501. doi: 10.1136/jmg-2023-109531
